# The winding road to health: A systematic scoping review on the effect of geographical accessibility to health care on infectious diseases in low- and middle-income countries

**DOI:** 10.1371/journal.pone.0244921

**Published:** 2021-01-04

**Authors:** Fleur Hierink, Emelda A. Okiro, Antoine Flahault, Nicolas Ray

**Affiliations:** 1 Institute of Global Health, Faculty of Medicine, University of Geneva, Geneva, Switzerland; 2 Institute for Environmental Sciences, University of Geneva, Geneva, Switzerland; 3 Population Health Unit, Kenya Medical Research Institute - Wellcome Trust Research Programme, Nairobi, Kenya; 4 Centre for Tropical Medicine and Global Health, Nuffield Department of Clinical Medicine, University of Oxford, Oxford, United Kingdom; 1. IRCCS Neuromed 2. Doctors with Africa CUAMM, ITALY

## Abstract

**Background:**

Geographical accessibility to healthcare is an important component of infectious disease dynamics. Timely access to health facilities can prevent disease progression and enables disease notification through surveillance systems. The importance of accounting for physical accessibility in response to infectious diseases is increasingly recognized. Yet, there is no comprehensive review of the literature available on infectious diseases in relation to geographical accessibility to care. Therefore, we aimed at evaluating the current state of knowledge on the effect of geographical accessibility to health care on infectious diseases in low- and middle-income countries.

**Methods and findings:**

A search strategy was developed and conducted on Web of Science and PubMed on 4 March 2019. New publications were checked until May 28, 2020. All publication dates were eligible. Data was charted into a tabular format and descriptive data analyses were carried out to identify geographical regions, infectious diseases, and measures of physical accessibility among other factors. Search queries in *PubMed* and *Web of Science* yielded 560 unique publications. After title and abstract screening 99 articles were read in full detail, from which 64 articles were selected, including 10 manually. Results of the included publications could be broadly categorized into three groups: (1) decreased spatial accessibility to health care was associated with a higher infectious disease burden, (2) decreased accessibility was associated to lower disease reporting, minimizing true understanding of disease distribution, and (3) the occurrence of an infectious disease outbreak negatively impacted health care accessibility in affected regions. In the majority of studies, poor geographical accessibility to health care was associated with higher disease incidence, more severe health outcomes, higher mortality, and lower disease reporting. No difference was seen between countries or infectious diseases.

**Conclusions:**

Currently, policy-makers and scientists rely on data collected through passive surveillance systems, introducing uncertainty on disease estimates for remote communities. Our results highlight the need for increasing integration of geographical accessibility measures in disease risk modelling, allowing more realistic disease estimates and enhancing our understanding of true disease burden. Additionally, disease risk estimates could be used in turn to optimize the allocation of health services in the prevention and detection of infectious diseases.

## 1. Introduction

Infectious diseases remain a major cause of global disease burden [1], especially in low resource settings where insufficient resources, inadequate infrastructures and poor access to health services impact disease outcomes [2, 3]. Timely access to health facilities can prevent further disease progression and as a result improves individual and public health outcomes [4]. However, large disparities in geographical (i.e. physical or spatial) accessibility persist because facilities are present at finite fixed locations while health needs vary across space and time, which potentially poses higher risks to remote communities [5, 6].

Poor physical accessibility to health facilities is a particularly important spatial aspect of disease control, as it delays or limits case detection through passive surveillance systems [4, 7, 8]. When distance to health care hinders accessibility, mild infections might develop into severe disease stages, potentially leading to suboptimal care outside the health system or even mortality. In addition to this, chances are that the infectious disease remains unnotified and silently infects more people, leading to unexpected outbreaks. Recent outbreaks, among which the COVID-19 pandemic and the Ebola outbreak in the Democratic Republic of Congo, have shown that our true understanding of disease estimates lags behind. Political instability, conflict, poor health systems, and remoteness hinder timely epidemiological surveillance [9, 10]. Substantial reporting delays are a result, leading to nationwide misrepresentations of disease estimates, allowing infectious diseases to silently continue their spread [10].

While the main global indicator of national progress towards infectious disease elimination is disease incidence rate, it has long been acknowledged that these rates cannot be directly measured through passive surveillance systems due to missed disease reporting along the patient pathway [11, 12]. Measuring the gap between notified disease cases and missed disease reports allows the extrapolation of more realistic disease estimates [4, 12]. Geographical accessibility to health care has previously been acknowledged as an important denominator of missed disease reporting and could therefore potentially serve as a correction factor for national and especially sub-national disease estimates [4, 12].

However, to our knowledge no review has systematically explored the literature to identify and discuss the findings on considering geographical accessibility in infectious disease research and more specifically on understanding true disease burden. Scoping reviews are a suitable methodology to capture research results from interdisciplinary fields and from a wide variety of literature sources [13]. The scoping review approach is therefore particularly suitable for our study.

Under the current COVID-19 pandemic there is a strong need for modelling techniques that account for underreporting introduced by inadequate surveillance capacity, to enable allocation of limited resources, such as testing services, and deployment of control strategies [9]. Outbreak preparedness, prevention, and control strategies strongly rely on a country’s health system and case detection capacity, which is generally assumed to be low in low- and middle-income countries (LMICs) due to weak health care systems and scarcity of human and financial resources [14]. Geographical accessibility models can capture important information on the spatial distribution of detection probability and might therefore be a strong indicator for realistic disease estimates [15–17].

The objective of this scoping review is therefore to evaluate the current state of knowledge on the effect of geographical accessibility on infectious diseases in LMICs. This review will also seek to capture the most important findings for researchers and health practitioners working on national disease reports, to potentially enable; 1) data correction measures along accessibility gradients, 2) targeting infectious disease interventions based on spatial accessibility information.

## 2. Methods

We followed the scoping review methodology recommended by Arksey and O’Malley [13]. We adhered to the PRISMA guidelines [18] (S1 Table).

### 2.1 Eligibility criteria

We targeted publications capturing measures or proxies of geographical accessibility in relation to infectious diseases. To cover the widest variety of available literature we used geographical accessibility in its broadest sense and did not specify certain measures of accessibility, such as time, distance or coverage. Peer-reviewed journal articles, conference papers, book chapters, short communications, and dissertations, either written in English, Dutch, or French, were included. We excluded articles studying high-income countries (based on the historical World Bank classification) [19], keeping only studies on LMICs. Articles focusing on the effect of spatial accessibility on non-communicable diseases, studies centered around animal disease outbreaks, publications studying the distance between patients as a matter of outbreak potential and those looking at genomic distance between isolates of pathogens, were also excluded. All publication dates were eligible.

### 2.2 Search strategy

The initial searches were executed in *PubMed* and *Web of Science* on March 4, 2019. New publications were checked until May 28, 2020. These searches comprised two main health concepts, namely geographical accessibility to health care and infectious diseases. These two concepts were subsequently subdivided into four major keyword groups: 1) accessibility, 2) geospatial analyses, 3) health care facilities, and 4) infectious diseases. All keyword categories were discussed between two authors (FH and NR), and preliminary formative literature searches were done on solely the identified keywords within a category, to check its accuracy and specificity. Accessibility keywords included *access*, *time*, and *barriers*, since it was expected that accessibility measures varied across publications. To ensure that publications captured spatial accessibility, and no other important aspects of access (e.g. financial accessibility or social accessibility), we included keywords describing the geographical dimension of the analyses: *geographic*, *geospatial*, *GIS*, *geographic information system*, or *spatial*. In addition, health facilities were described by the keywords *hospital*, *health centers*, *health care*, and *health facilities*. Since this review is not specified for one infectious disease, this category was captured by the keywords *epidemic*, *outbreak*, and *infectious diseases*.

With *Pubmed*, Medical Subject Headings (MeSH) terms were used in addition to the identified keywords (see S1 File for full search strategy). Secondary search techniques were used by applying snowball search, and through *Google Scholar* with the following keyword sets: *travel time and infectious diseases*, *barriers to health care access and infectious diseases*, and *geographical access and infectious diseases*.

### 2.3 Publication selection: Title, abstract, and full article screening

All searched publications were exported to tabular format in Microsoft Excel version 16.21.1 [20]. Duplicates were removed. Titles and abstracts were independently screened by FH and NR for conformity with in- and exclusion criteria. Mismatches in decisions were openly discussed by the two authors and, in case of doubt, always included for full article reading. A final set of publications was produced by consensus.

### 2.4 Charting the data

All selected publications were read by FH, and publication details were extracted accordingly. The extracted variables included; authors, first author affiliation, publication year, study design, the country of study conduction, sub-national region, study year, study population, infectious disease, geographical accessibility metric, and direction of association between access and infectious disease. Data analysis and visualization was carried out in Quantum Geographical Information System (QGIS version 3.12) [21] and R (R version 3.5.2) [22].

## 3. Results

### 3.1 Overview of literature search

**Fig 1:**
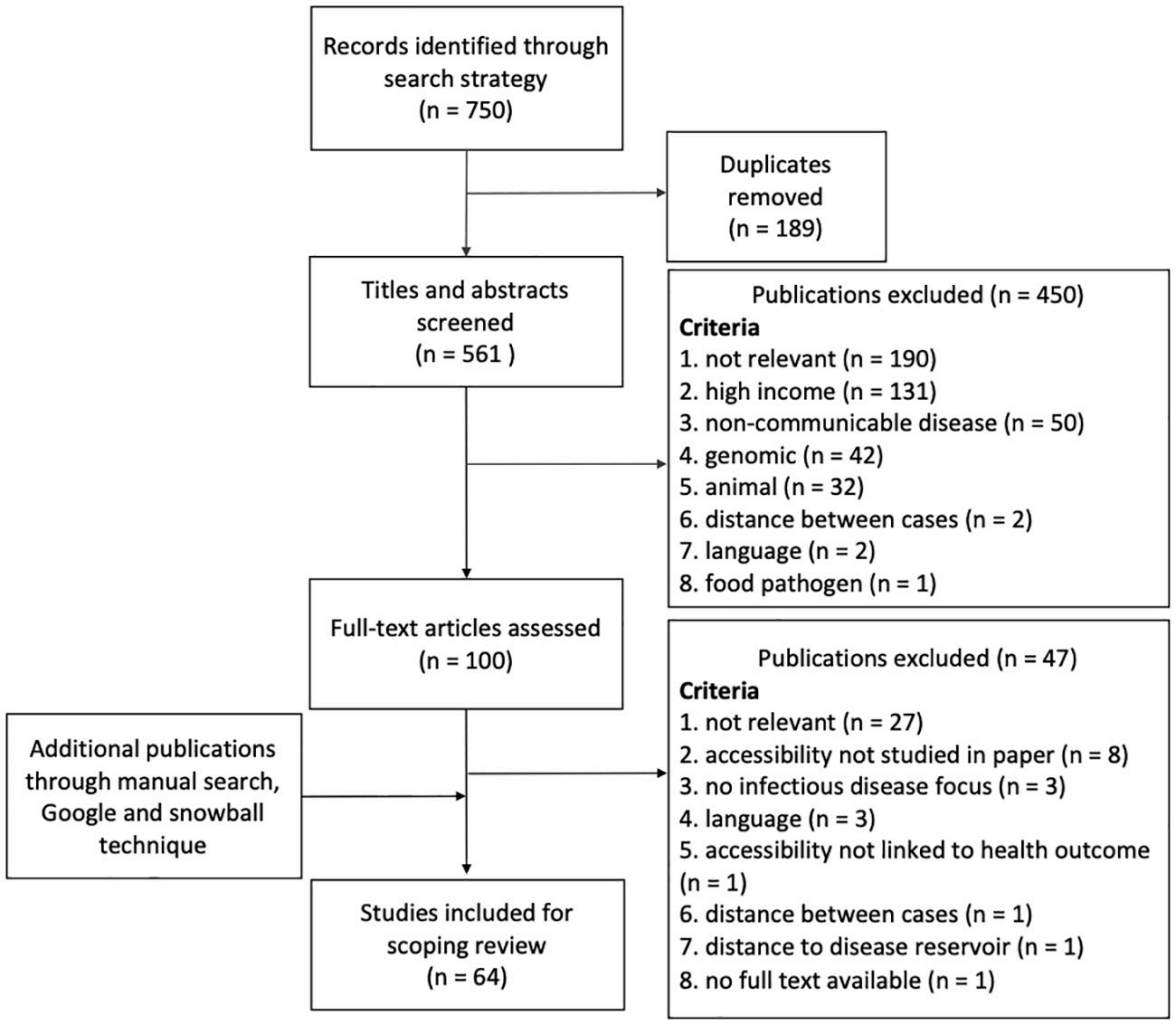
Overview of iterative article selection process and selected articles at each step.

The search strategy yielded 560 unique publications. After title and abstract screening, 99 full articles were read and assessed on eligibility criteria. We finally included 64 research papers, including 10 manually searched articles (Fig 1). Unfortunately, 4 articles had to be excluded because of language restrictions, namely one in Japanese [23], two in Portugese [24, 25], and one in Spanish [26]. The data used in the papers ranged from 1987 to 2018. Most studies used retrospectively collected health data and linked this to subnational administrative units to calculate the accessibility metric. Full details on the selected articles are available in S2 Table.

### 3.2 Infectious diseases

Human Immunodeficiency Virus (HIV)/Aids was the most studied infectious disease in relation to accessibility (n = 14), followed by tuberculosis (TB) (n = 8), cholera (n = 5), dengue (n = 5), and measles (n = 2) and malaria (n = 2). More general measures of the impacts of infectious diseases comprised disease burden (n = 4), child mortality (n = 4), and childhood vaccination (n = 3). Other studied diseases included Ebola, Nipah, sleeping sickness, tetanus, typhoid fever, and dog-bites (S3 Table).

### 3.3 Geographical patterns of studies

**Fig 2:**
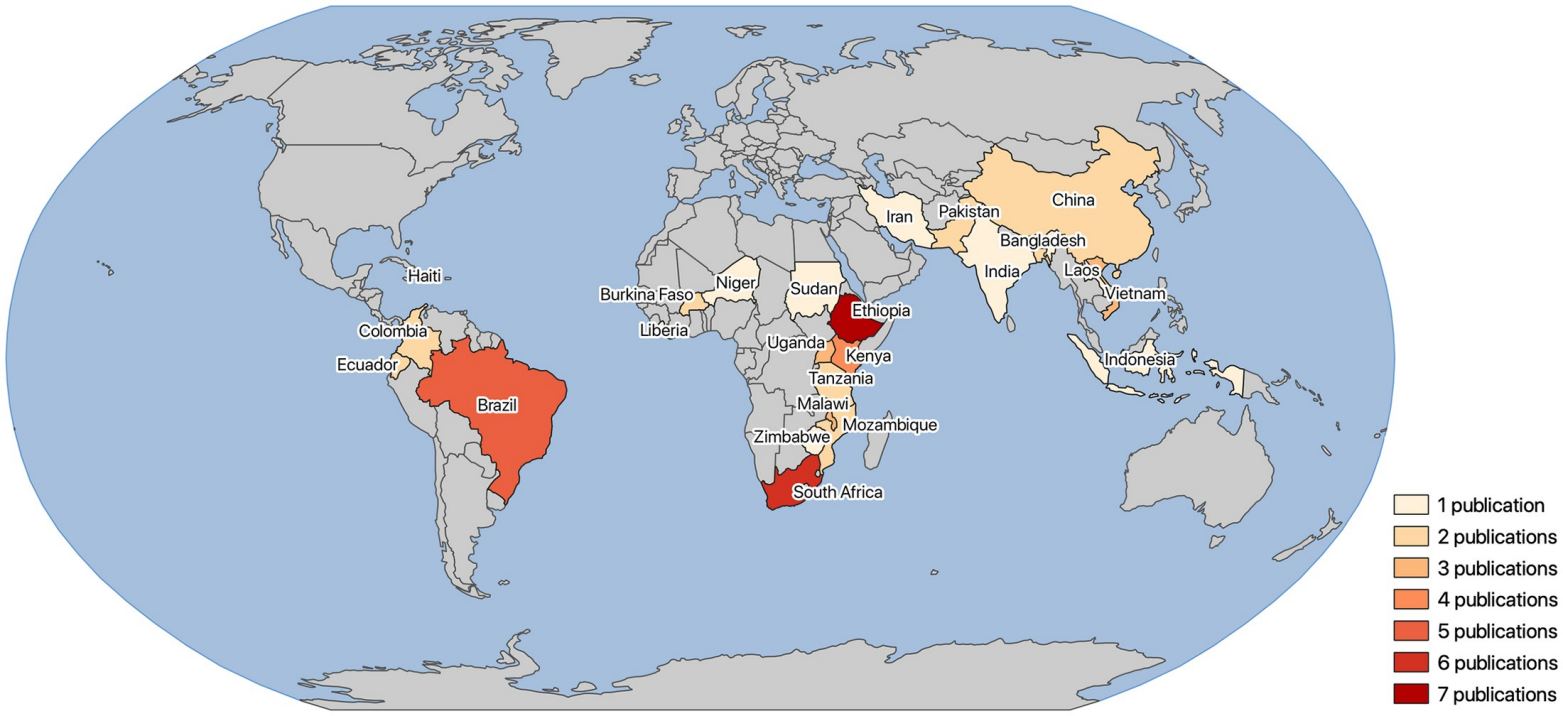
Countries where geographical accessibility studies were conducted in relation to infectious diseases. Colors indicate the number of publications per country. Map in Figure was created using shapefiles sourced from Natural Earth.

In general, studies were carried out in 24 unique countries. The majority of the articles considered studies conducted on the African continent (n = 33), followed by South America (n = 9), and Asia (n = 8) (Fig 2, S4 Table). Of all countries combined, most studies were conducted in Ethiopia (n = 7), South Africa (n = 6), Brazil (n = 5), and Kenya (n = 4) (Fig 2, S4 Table). Some articles studied multiple countries, larger regions (e.g. Sub-Saharan Africa), or had a global perspective and could therefore not be linked to country borders (n = 6). When stratifying the studied infectious diseases by country, it became apparent that HIV/Aids articles mainly considered Southern African countries, namely South Africa (n = 3), Mozambique (n = 2), Malawi (n = 2), Zimbabwe (n = 1), and the full Southern Africa region (n = 1) (S1 Fig). Cholera-related research included Haiti (n = 2), Iran (n = 1), Tanzania (n = 1), and Uganda (n = 1). Dengue research in relation to accessibility was carried out in Colombia (n = 2), Brazil (n = 1), Ecuador (n = 1), India (n = 1).

### 3.4 Consistency of accessibility measures

Geographical accessibility measures were highly variable across studies. Most studies used a distance metric in meters or kilometers to capture health care accessibility (n = 29) (S2 Table). However, within this metric there was no consistency in how distance was measured. Studies reported either distance by road, Euclidean distance, or did not report any description on measurement details. The second largest accessibility measure used was travel time (n = 9) (S2 Table). Other categories as a proxy of accessibility included, but were not limited to, number of health facilities in a sub-national district (n = 3), a combination of travel time and distance (n = 2), immunization coverage as an indicator of primary care reach out (n = 1), and antenatal care coverage as a proxy for access to measles immunization (n = 1) (S2 Table).

### 3.5 Geographical accessibility to health care and infectious diseases

Out of the 64 included studies, 52 statistically analyzed the association between geographical accessibility and the impact on the studied infectious disease (S2 Table). Since study results were heterogeneously presented across studies, the direction of the association could not be unambiguously interpreted for all studies.

In general, the results of the included publications could be broadly categorized into three groups; (1) decreased geographical accessibility to health care was associated with a higher infectious disease burden in remote communities, (2) decreased accessibility was associated with lower disease reporting, and (3) the occurrence of an infectious disease outbreak affected health care accessibility.

#### 3.5.1 Poor geographical accessibility and increased disease burden

Poor spatial accessibility to health care was found to be associated with higher disease incidence, more severe health outcomes, and higher mortality in the majority of studies. No difference was seen between countries or infectious diseases. Measles case fatality rates in Ethiopia for instance, were found to increase from 0.62% in close proximity of the hospital to more than 20% for communities more than 20 km away of the health facility [27]. In Kenya, researchers found that the incidence of malaria increased with a factor 2.5 as travel time increased from 10 minutes up to 2 hours to the nearest health facility [6]. Diarrheal mortality in children living in Pakistan was also significantly associated with travel time, as children who had to travel more than 1 hour to a health facility, had 3.6 higher odds on mortality than children travelling less than 1 hour [28]. A study conducted on febrile illnesses in children in Tanzania showed that children living more than 3 hours from a hospital were twice as likely to die from their condition than children living less than 3 hours from a facility [29]. In Bangladesh, the probability of attending care for Nipah case fatalities, also markedly decreased with distance from the hospital. Eighty-two percent of case fatalities living less than 10 km visited a health facility, with this figure dropping to 54% for case fatalities living more than 50 km away [30].

In addition to the findings presented above, univariate regression analysis on cholera data from Uganda showed statistically significant associations between distance to the nearest health facility and cholera incidence [31]. In Haiti, cholera was also found to lead to more severe outcomes and higher mortality rates in remote communities than in communities with better access to health care [7]. Another study that looked at tetanus in Indonesia found that distance to the hospital significantly differed between tetanus survivors and fatalities. Patients who had died from tetanus travelled on average more than 29 km whereas survivors traveled about 6.5 km [32]. In rural South Africa, HIV/TB patients were found to have a higher risk of dying when living further than 5 km from the health facility than the patient group living within 5 km of the health center [33].

However, some other studies presented protective associations between distance from health facilities and infectious disease incidence. In a study carried out by Telle et al. [34], it was found that dengue cases in Delhi, India were significantly lower with increased distance from the sentinel hospital. This same association was supported in a study on HIV in East Zimbabwe, were higher self-reported distances to treatment facilities were found in low prevalence clusters [35]. In a study on cholera in Dar Es Salaam, Tanzania, distance to the nearest clinic or a major road was also not statistically related to cholera incidence [36]. Shorter distances to local health centers was also found to contribute to the spread of cholera in Chabahar, Iran, although this might have been due to increased transport in this region and higher population mobility [37].

Infectious diseases were also found to be more severe upon first presentation at a health facility when living remotely. In Uganda, sleeping sickness patients living in close proximity to a health facility were more likely to present in an early stage of the disease than patients living further away [38]. In addition, rabies deaths in Pakistan were disproportionally higher in the health center with the longest reported travel times among dog-bite patients. Late stage presentation of cases and the absence of appropriate vaccination were likely to have contributed to this higher mortality [39].

Besides travel time to health facilities, more general measures of mobility were also shown to describe disease patterns. In Malawi for instance, mean travel times to the nearest public transport contributed the most to describing HIV prevalence at the district level [40]. These findings were supported by a study conducted in rural South Africa, where households owning a motorized vehicle had lower HIV/TB mortality rates [33]. In Haiti, case fatality rates of cholera were found to be higher when the mode of transport was by foot [7].

#### 3.5.2 Poor geographical accessibility and decreased disease reporting

In addition to the effect of accessibility on higher disease burden, it also became apparent that accessibility is strongly linked to disease reporting. Hospitalization rates and utilization of infectious disease testing services were generally found to dramatically decrease with distance. Delayed or missed disease reporting minimizes the true understanding of infectious disease burden and complicates the localization of disease outbreaks, which are needed for targeted interventions and informed implementation of health programs [10, 41, 42]. In Bangladesh, it was estimated that only 52% of Nipah outbreaks were detected through passive surveillance systems and that the number of missed outbreaks increased with distance from the surveillance hospital [30]. This finding was supported by MacPherson et al. [43], who found that the TB case notification rate halved for every 3.2–3.5 fold increase in distance from the nearest TB clinic. In a study carried out by Etyang et al. [44], there was a significant decrease in infectious- and parasitic disease related hospital admissions for every 5 km increase in distance from the hospital. In a study conducted by Poletti et al. [27] it was estimated that measles hospitalizations in Ethiopia dropped from 31.0% in close proximity of a health facility to 5.7% at sites located 30 km from the hospital. The number of missed severe measles cases also significantly increased with distance from the hospital [27]. In Tanzania, febrile illness related hospital admissions declined from 125/1000 children in the catchment population in less than 3 hours of the catchment of the hospital to 25/1000 when living more than 3 hours from the hospital [29]. Tuberculosis incidence in Sheka Zone, Ethiopia was found to significantly increase in the presence of health facility in the respective district [45]. In addition, in Uganda the number of sleeping sickness cases detected significantly decreased with distance from the hospital [38]. Furthermore, a study conducted to TB incidence in Vietnam showed that the distance to the nearest TB treatment facility presents a negative effect on the notified TB incidence, with notified cases decreasing by a factor of 0.87 for every km increase in distance [41]. Similar results were found in a study where healthcare seeking for infectious diseases in Bangladesh declined with increasing distance from health facilities [46]. In western Kenya, clinic visits were also found to be influenced by distance from health facilities, where every 500 m increase in distance from the health facility caused a linear decline in clinic visits up to 4 km, after which the association stabilized [47]. The utilization of HIV testing services in rural Mozambique was also influenced by distance to the service, as HIV testing decreased with distance. Expanding HIV-related services decreased the influence of spatial barriers on service utilization and decreased spatial variability [48].

The results above address the fact that disease notification probability decreases with distance from health facilities, causing underrepresentation of clinical data in settings with poor health care access. In a recent publication, geographical accessibility data has been used to model the probability of passive malaria case detection in Burkina Faso, to eventually predict the spatial distribution of the disease [4]. This highlights the timeliness and importance of accessibility modelling in infectious disease studies.

#### 3.5.3 Infectious disease outbreaks and accessibility

Besides the effects of accessibility on infectious diseases, the occurrence of an infectious disease outbreak was also found to have substantial effects on health care accessibility. These results are particularly relevant in light of the current COVID-19 pandemic. In a study carried out after the 2014 Ebola outbreak in Liberia, qualitative research showed that 57% of interviewed households experienced difficulties in accessing health care during the epidemic. Urban areas seemed to be affected more than rural communities, with over 65% of the urban respondents reporting challenges in accessing health care and only 20–30% of the urban respondents being able to receive care when seeking [49]. In a recent publication from Hulland et al. [50], geographical health care accessibility rasters for 43 African countries were used as vulnerability component in the risk assessment of viral hemorrhagic fever outbreaks across the African continent. Findings showed that accessibility was low in some areas of outbreak potential and indicated regions for targeted health system optimization, so outbreak preparedness could be strengthened [50]. Another study, carried out by Casas and Delmelle [51], looked at health care utilization during a dengue outbreak in Colombia and showed that patients suffering from dengue were willing to travel further for a specific set of health facilities. Over 90% of the dengue patients did not travel to the closest health facility for treatment. In addition, there was no distance decay association found between dengue fever diagnosis and distance from health facilities during the epidemic [51]. A study carried out in the same study setting additionally showed that travel times for dengue patients during the outbreak were much longer than estimated travel times, implying that patients decided to travel further than the nearest health facility [52]. When solely focusing on the effect of infectious diseases on geographical accessibility and not utilization, it was found that geographical accessibility indeed can decrease during outbreaks, however this effect varies spatially depending on the health facility of choice by the majority of the patients.

## 4. Discussion

### 4.1 General results

Our scoping review process identified 64 articles describing accessibility in relation to infectious diseases in LMICs. The majority of studies were conducted on the African continent and focused on HIV/Aids, access to antiretroviral therapy (ART) and tuberculosis. The fact that these infectious diseases are the ones with the highest global burden, is likely to be the reason of this representation in scientific literature [1]. Interestingly, our search did not yet cover any articles on the influence of physical accessibility on COVID-19 detection. However, some researchers already addressed concerns about the health system response and reaction capacity in LMICs countries most at risk [9, 15–17].

Fifty-two articles statistically analyzed the impact of spatial accessibility on an infectious disease. It became apparent that the effects of spatial accessibility to health care on infectious diseases were homogeneous among countries and infectious diseases, with lower disease reporting, higher predicted disease incidence, more severe health outcomes, and increasing mortality rates with decreasing geographical access to health care. It is likely that similar effects are seen for COVID-19 and it is therefore of high importance to consider these findings in light of the current outbreak.

### 4.2 The protective efficacy of improved access on infectious diseases

Many of our selected literature highlighted the negative impacts of poor accessibility on infectious disease outcomes but improving spatial accessibility has also proven to be effective in reducing infectious disease impacts [6]. In a study carried out by Gerberry et al. [53], geospatial targeting of antiretroviral therapy based on incidence, was shown to prevent approximately 40% more HIV infections, compared to using an egalitarian distribution. In addition, O’Meara et al. [6] have shown that if all children in a rural district of Kenya would live within a 1-hour walking distance of a health facility, an additional 500 malaria hospitalizations could have been effectively averted. Furthermore, a significant improvement in the use of HIV testing services was seen with increased spatial accessibility to these services [48]. Completed child immunization was also significantly higher in regions located within 1-hour of a health facility compared to more distant districts [54]. These findings stress the efficacy of increased spatial accessibility on the prevention and reduction of infectious disease impacts in remote communities.

### 4.3 Optimizing health services by targeting infectious diseases

Optimization of accessibility to care is important for improving health outcomes on all aspects of health [5]. However, health services that provide care in situations with an acute need are most important to be efficiently targeted to best serve the population [55, 56]. Conversely, infectious diseases with an acute need for treatment were particularly underrepresented in the selected literature covering spatial accessibility. Despite the need for prompt administration of vaccines and thus adequate access to health care, diseases like tetanus or rabies were only covered in one study. In a paper on access to vaccination sites for animals, the importance of spatial accessibility in the battle against the transmission of rabies in Brazil was stressed [57]. It was put forward that in order to reduce transmission and health risks, the allocation of health services providing vaccines should be based on spatial accessibility information [57]. In infectious diseases like rabies, which is a 100% lethal disease, timely access to health care providing post-exposure prophylaxis can make a direct difference in mortality risk for the patient [58, 59]. However, since resources in endemic countries are often limited, targeted distribution of vaccines is key, this also greatly applies to the distribution of testing services for COVID-19. The optimization and targeted allocation of prevention measures in the fight against infectious diseases could be based on enhancing population coverage so that the largest population proportion is covered under a certain travel time-based catchment [60]. However, health services can also be optimized based on the spatial variation in infectious disease risks, as was also presented by Gerberry et al. [53] and Hulland et al. [50]. Since infectious diseases often show spatial clustering, allocating services to regions with highest risks could subsequently lead to a targeted reduction in incidence and increased preparedness in case of outbreaks [50]. Improving spatial access to care in areas of high disease burden could also result in earlier detection of disease cases and has proven to result in decreased intensity of some infectious diseases in Uganda [61]. We gather from the selected literature that research in the field of accessibility and infectious diseases has been recently evolving, with vast improvements of infectious disease estimates integrating accessibility modelling efforts.

### 4.4 Moving towards accessibility-driven correction methods

Understanding true disease burden is crucial for ongoing epidemic risk assessments. Over the last years we have seen that our capacity to timely detect and respond to outbreaks still lags behind [10, 62]. Identifying important factors that can explain large scale variation in underreporting, such as spatial accessibility, can support the correction of sub-national disease numbers, collected through passive surveillance systems [4]. Since geographical accessibility maps for health care, especially those based on travel time, manifest important information on our capacity to detect disease cases, disease numbers could be corrected along accessibility gradients as has been put forward in previous research [4, 11, 30]. Knowing the proportion of a population within a certain catchment of a health facility that is able to reach a facility for disease reporting, can support disease extrapolation or disease notification probabilities in larger travel time catchments [4]. Accessibility-driven correction methods allow more realistic infectious disease modelling and can increase our understanding of infections that are missed through passive disease surveillance. This information can eventually improve our capacity in early detection of infectious diseases.

### 4.5 Future research

The information presented in sections 4.3 and 4.4 suggest that future research should increasingly integrate accessibility-driven correction methods together with infectious disease-based health service optimization. This can be done by using spatial accessibility measures; 1) as a proxy for the capacity to detect infectious diseases in remote communities and subsequently correct for this potential underreporting, and 2) to identify regions with poor access to health care and eventually use the extrapolated infectious disease numbers from step one, to optimize accessibility in a targeted way. Additionally, our results reflect the use of a wide variety of geographical accessibility measures, among which distance to health facilities was the largest category. However, distance measures might not account for physical barriers (e.g. rivers, mountains, protected nature reserves, etc.) experienced by patients travelling to health facilities and therefore overestimate geographical accessibility to health care. We would therefore recommend future research to apply more realistic measures of accessibility, such as travel time [60].

### 4.6 Limitations of the study

This article presents the first results of a scoping review study to the influence of geographical accessibility to healthcare on infectious diseases in LMICs. However, there were several limitations identified throughout the study. Firstly, only articles in English, French, and Dutch were included, potentially leading to the exclusion of some papers. Secondly, the search strategy was carried out in *PubMed* and *Web of Science*, likely leaving out some interesting results published in journals not covered by these databases. However, we attempted to bridge this gap by using the snowball technique and manual identification of relevant references. Thirdly, even though we carefully considered the search terms included in our strategy, our search potentially has missed some keywords. For the bigger keyword groups we decided to include keywords that might also reflect specific synonyms (e.g. “health facilities” instead of “clinics”), but this might have not covered all literature available. Yet, we feel confident that the risk of missing important literature was minimized by applying hand search and snowball techniques in the available literature.

## 5. Conclusion

The results of this study have shown that (1) decreased geographical accessibility to health care was associated with a higher infectious disease burden in remote communities, (2) decreased accessibility was associated with lower disease reporting, and (3) the occurrence of an infectious disease outbreak affected health care accessibility. These results reflect that we lack a clear understanding of infectious disease estimates and burden in regions with poor spatial health care access. Reliable estimates of infectious diseases are needed to enable risk assessments for guided allocation of preventive measures and targeted disease containment. Our results highlight a strong need for integration of spatial accessibility measures in infectious disease risk modelling, allowing more realistic disease estimates. The results presented here provide a platform for moving towards accessibility-driven disease correction measures, which enables more realistic disease estimates and could subsequently lead to earlier detection of outbreaks. Additionally, disease risk estimates could be used in turn to optimize the allocation of health services in the prevention and detection of infectious diseases.

## Supporting information

S1 Fig Number of papers stratified for country and infectious disease. (TIF)

S1 Table Preferred reporting items for systematic reviews and meta-analyses extension for scoping reviews (PRISMA-ScR) checklist. (DOCX)

S2 Table Overview of data extracted from included articles. (DOCX)

S3 Table Number of papers per disease category. (DOCX)

S4 Table Number of papers per country where studies were conducted. (DOCX)

S1 File Database search terms. (DOCX)
