# Supplementary figures and images for: The winding road to health: A systematic scoping review on the effect of geographical accessibility to health care on infectious diseases in low- and middle-income countries
Source: PLoS One. 2021 Jan 4;16(1):e0244921. doi: 10.1371/journal.pone.0244921 (PMC7781385; doi:10.1371/journal.pone.0244921)

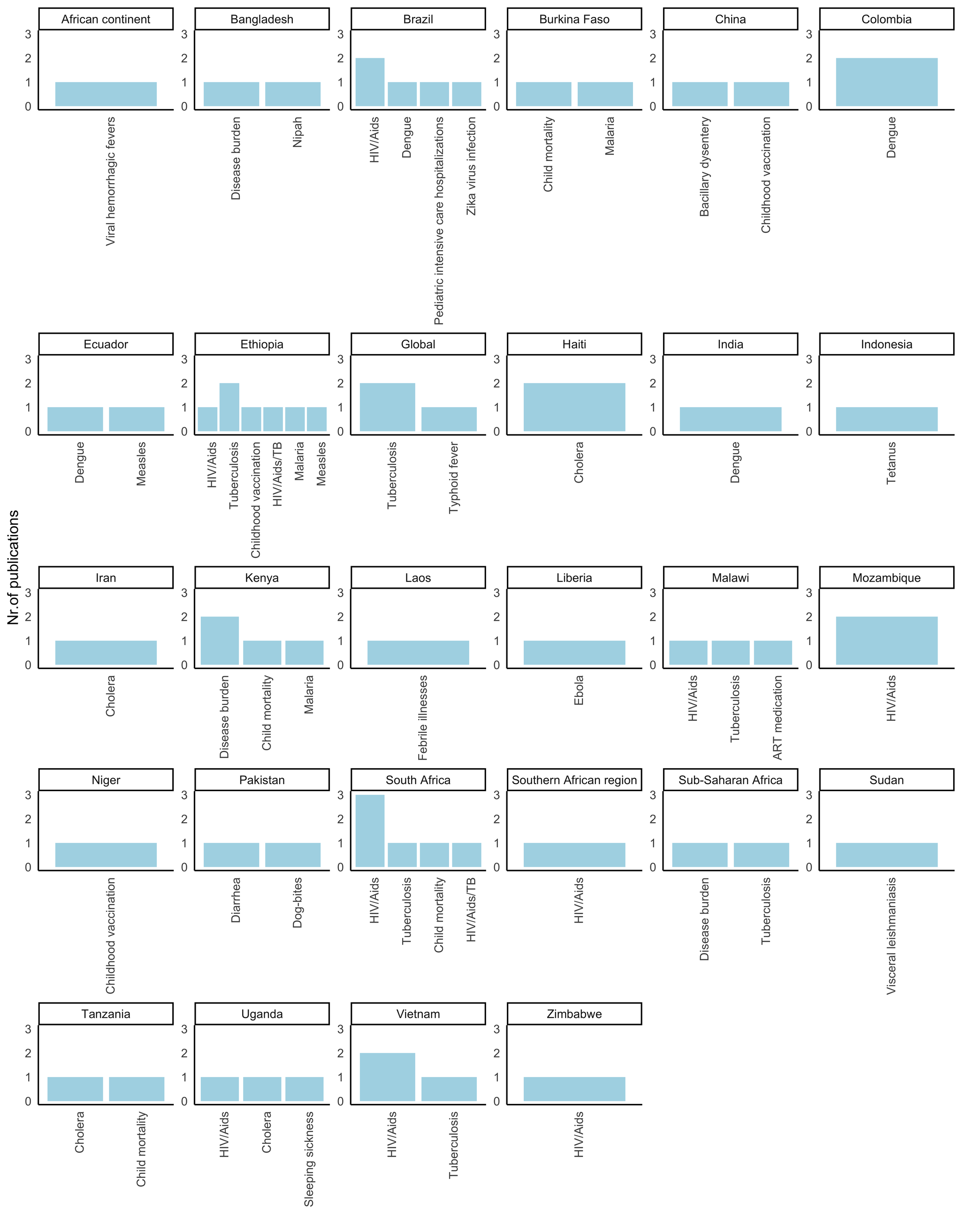

Supplement: S1 Fig — (TIF) [file pone.0244921.s001.tif]
