# Supplementary material for: The winding road to health: A systematic scoping review on the effect of geographical accessibility to health care on infectious diseases in low- and middle-income countries
Source: PLoS One. 2021 Jan 4;16(1):e0244921. doi: 10.1371/journal.pone.0244921 (PMC7781385; doi:10.1371/journal.pone.0244921)
Supplement: S2 Table — (DOCX) [file pone.0244921.s003.docx]

| **Source**  **S2 Table. Overview of data extracted from included articles.** | **Title** | **Author** | **Journal** | **Statistically tested** | **Study design** | **Country** | **Subnational region** | **Geographical aggregation** | **Time period** | **Infectious disease** | **Accessibility variable** | **Unit accessibility variable** |
| --- | --- | --- | --- | --- | --- | --- | --- | --- | --- | --- | --- | --- |
| Database search | Role of health determinants in a measles outbreak in Ecuador: a case-control study with aggregated data | (Rivadeneira, Bassanesi, and Fuchs 2018) | BMC Public Health. 2018 Feb 20 | yes | Case-control | Ecuador | Country wide | Parishes (smallest region adm) | 2011-2012 | Measles | Antenatal care coverage | % |
| Database search | Mapping malaria risk using geographic information systems and remote sensing: The case of Bahir Dar City, Ethiopia | (Minale and Alemu 2018) | Geospatial health (2018) | yes | Risk assessment model | Ethiopia | Bahir Dar City | City | unclear | Malaria | Distance from health facility | in meters |
| Database search | Barriers to initiating tuberculosis treatment in sub-Saharan Africa: a systematic review focused on children and youth | (Sullivan, Esmaili, and Cunningham 2017) | Global health action, (2017) | no | Systematic review | Sub-Saharan Africa | - | - | - | Tuberculosis | Infrastructure as a barrier to TB treatment | |
| Database search | Heterogeneity of distribution of tuberculosis in Sheka Zone, Ethiopia: drivers and temporal trends | (Shaweno et al. 2017) | International Journal of Tuberculosis and Lung Disease (2017) | yes | Retrospective | Ethiopia | Sheka Zone | Kebele (smallest region adm) | 2010-2014 | Tuberculosis | Health facility availibility | |
| Database search | Spatial patterns of HIV prevalence and service use in East Zimbabwe: implications for future targeting of interventions | (Schaefer et al. 2017) | Journal of the International AIDS Society (2017) | yes | Open cohort study | Zimbabwe | Manicaland, East Zimbabwe | 3 districts in Manicaland | 2012-2013 | HIV/Aids | distance to ART, HTC, health facility and hospital | in km |
| Database search | The hidden burden of measles in Ethiopia: how distance to hospital shapes the disease mortality rate | (Poletti et al. 2018) | BMC Med. 2018 Oct 18 | yes | Retrospective | Ethiopia | South West Shoa Zone, Oromia Region | Kebele (smallest region adm) | 2013-2017 | Measles | distance to hospital | in km |
| Database search | Spatial distribution of extensively drugresistant tuberculosis (XDR TB) patients in KwaZulu-Natal, South Africa | (Kapwata et al. 2017) | PLoS One. 2017 Oct 13 | no | Retrospective | South Africa | KwaZulu-Natal | Districts | 2011-2014 | Tuberculosis | distance to closest HF | in km |
| Database search | Potential versus revealed access to care during a dengue fever outbreak outbreak | (Casas, Delmelle, and Delmelle 2017) | Journal of Transport & Health (2017) | yes | Retrospective | Colombia | Cali | Communes | 2010 | Dengue | Potential travel time | in minutes |
| Database search | Identifying cholera "hotspots" in Uganda: An analysis of cholera surveillance data from Identifying cholera "hotspots" in Uganda: An analysis of cholera surveillance data from 2011 to 2016 | (Bwire et al. 2017) | PLoS Negl Trop Dis. 2017 Dec 28 | yes | Retrospective | Uganda | Country-wide | Districts | 2011-2016 | Cholera | Distance from district centroid to nearest hospital | linear distance |
| Database search | The Spread of Dengue in an Endemic Urban Milieu–The Case of Delhi, India | (Telle, Vaguet, Yadav, Lefebvre, Daudé, et al. 2016) | PLoS one (2016) | yes | Retrospective | India | Delhi | City | 2008 to 2010 | Dengue | Distance to the nearest sentinel hospital | in meters |
| Database search | Geographic Distribution and Mortality Risk Factors during the Cholera Outbreak in a Rural Region of Haiti, 2010-2011 | (Page et al. 2015) | PLoS Negl Trop Dis. 2015 Mar 26 | yes | Community based study | Haiti | Nord department | Districts | 2011 | Cholera | Self-reported distance, remoteness, mode of transport | - |
| Database search | Using geospatial modeling to optimize the rollout of antiretroviral-based pre-exposure HIV interventions in Sub-Saharan Africa | (Gerberry et al. 2014) | Nat Commun. 2014 Dec 2 | no | Geospatial modelling and optimization techniques | South Africa | Country-wide | Provinces |  | HIV/Aids | No specific variable | - |
| Database search | Analyzing spatial clustering and the spatiotemporal nature and trends of HIV/AIDS prevalence using GIS: the case of Malawi, 1994-2010 | (Zulu, Kalipeni, and Johannes 2014) | BMC Infect Dis. 2014 May 23 | yes | Geospatial modelling and regression | Malawi | Country-wide | Districts | 1995-2012 | HIV/Aids | Distance to roads | in km |
| Database search | Tuberculosis in Tropical Areas and Immigrants | (Zammarchi, Bartalesi, and Bartoloni 2014) | Mediterr J Hematol Infect Dis. 2014 Jun 1 | no | Review | Global | - | - |  | Tuberculosis | - | - |
| Database search | Spatial and social inequities in HIV testing utilization in the context of rapid scale-up of HIV/AIDS services in rural Mozambique | (Yao, Agadjanian, and Murray 2014) | Health Place. 2014 Jul | yes | Longitudinal study | Mozambique | Gaza province (rural areas) | Districts | 2006, 2009, 2011 | HIV/Aids | Distance to nearest testing clinic | in km |
| Database search | Socio-Economic Factors of Bacillary Dysentery Based on Spatial Correlation Analysis in Guangxi Province, China | (Nie et al. 2014) | PloS one (2014) | yes | Spatial autocorrelation | China | Guangxi province | County level | 2009-2010 | Bacillary dysentery | Number of beds | - |
| Database search | Determinants of the risk of dying of HIV/AIDS in a rural South African community over the period of the decentralised roll-out of antiretroviral therapy: a longitudinal study | (Mee et al. 2014) | Global Health Action, 2014 | yes | Longitudinal cohort study | South Africa | Bushbuckridge sub-district of Ehlanzeni municipality of Mpumalanga province in South Africa | | 2007-2010 | HIV/Aids/TB | Road distances to Bhubezi CHC | in km |
| Database search | The changing landscape of public health in sub- saharan Africa: Control and prevention of communicable diseases needs rethinking | (Mboera et al. 2014) | Onderstepoort Journal of Veterinary Research (2014) | no | Review | Sub-Saharan Africa | - | - |  | Disease burden | - | - |
| Database search | Spatiotemporal clustering, climate periodicity, and social-ecological risk factors for dengue during an outbreak in Machala, Ecuador, in 2010 | (Stewart-Ibarra et al. 2014) | BMC Infect Dis. 2014 Nov 25 | yes | Retrospective | Ecuador | Machala | Neighbourhood level | 2010 | Dengue | Average distance to the central hospital | in km |
| Database search | Burden of disease in adults admitted to hospital in a rural region of coastal Kenya: an analysis of data from linked clinical and demographic surveillance systems | (Etyang et al. 2014) | Lancet Global Health (2014) | yes | Retrospective | Kenya | Kilifi District | Household | 2007-2012 | Disease burden | Distance to hospital | in km |
| Database search | Geographic Variation in Access to Dog-Bite Care in Pakistan and Risk of Dog-Bite Exposure in Karachi: Prospective Surveillance Using a Low-Cost Mobile Phone System. | (Zaidi et al. 2013a) | PLoS neglected tropical diseases (2013) | yes | Prospective cohort study | Pakistan | Karachi | Point locations | 2009-2011 | Dog-bites | Travel time to ER | in minutes |
| Database search | Model of cholera dissemination using geographic information systems and fuzzy clustering means: Case study, Chabahar, Iran | (Pezeshki et al. 2012) | Public Health. 2012 Oct | yes | Retrospective | Iran | Chabahar | Districts | 1997-2006 | Cholera | distance to the local health centre and distance to the district health centre | in km |
| Database search | A new analytical framework of 'continuum of prevention and care' to maximize HIV case detection and retention in care in Vietnam | (Fujita et al. 2012) | BMC Health Serv Res. 2012 Dec 29 | yes | Literature review and analytical framework | Vietnam | National | Districts | January 1990-August 2011 | HIV/Aids | Service density (nr of facilities/per district) | nr of facilities per district |
| Database search | Four Ways Geographic Information Systems Can Help to Enhance Health Service Planning and Delivery for Infectious Diseases in Low-Income Countries | (Brijnath and De Souza 2012) | Journal of health care for the poor and underserved (2012) | yes | Commentary | Global | - | - |  | Typhoid fever | Number of treatment facilities | |
| Database search | It’s a long, long walk: accessibility to hospitals, maternity and integrated health centers in Niger | (Blanford et al. 2012) | International journal of health geographics (2012) | yes | Retrospective | Niger | National | Subdistricts | Not specified | Childhood vaccination | Travel time to health facilties | in time |
| Database search | Geographic influences on sexual and reproductive health service utilization in rural Mozambique | (Yao et al. 2012) | Appl Geogr. 2012 Mar | yes | Retrospective | Mozambique | Chibuto, Chokwe, Guija, Mandlakaze | Districts | 2006 | HIV/Aids | Distance to health facilities | in km |
| Database search | Cholera Epidemic in Haiti, 2010: Using a Transmission Model to Explain Spatial Spread of Disease and Identify Optimal Control Interventions | (Tuite et al. 2011) | Ann Intern Med. 2011 May 3 | no | Retrospective | Haiti | National | Regional | 2010-2014 | Cholera | Available vaccines | - |
| Database search | Expanding HIV Testing Efforts in Concentrated Epidemic Settings: A Population-Based Survey from Rural Vietnam | (Pharris et al. 2011) | PLoS One. 2011 Jan 11 | yes | Cross-sectional survey | Vietnam | FilaBavi, Bavi district | Districts | April-May 2007 | HIV/Aids | Place of residence with regards to the district hospital | |
| Database search | Young and vulnerable: Spatial-temporal trends and risk factors for infant mortality in rural South Africa (Agincourt), 1992-2007 | (Sartorius et al. 2010) | BMC Public Health. 2010 Oct 26 | yes | Prospective | South Africa | Bushbuckridge, Aginocourt | Districts | 1992-2007 | Child mortality | Distance to health facilities | in km |
| Database search | Informal Urban Settlements and Cholera Risk in Dar es Salaam, Tanzania | (Penrose et al. 2010) | PLoS Neglected Tropical Diseases (2010) | yes | Retrospective | Tanzania | Dar es Salaam | Municipality | 2006-2008 | Cholera | Distance to the nearest cholera treatment facility | in meters |
| Database search | Geospatial distribution and determinants of child mortality in rural western Kenya 2002–2005 | (Ombok et al. 2010) | Tropical Medicine & International Health (2010) | yes | Prospective | Kenya | Bondo and Siaya districts, rural parts of Nyanza Province | Villages | 2002-2005 | Child mortality | Distance to health facility | in meters |
| Database search | Characteristic tetanus infection in disaster-affected areas: case study of the Yogyakarta earthquakes in Indonesia | (Sutiono et al. 2009) | BMC Res Notes. 2009 Mar 6 | yes | Retrospective | Indonesia | Yogyakarta | Patient location | 2006 | Tetanus | Distance to health facility by road, categorized as under 15 and more than 15km | in km |
| Database search | Surveillance of mother-to child HIV transmission: socioeconomic and health care coverage indicators | (Barcellos et al. 2009) | Revista de saude publica (2009) | yes | Retrospective ecological study | Brazil | Porto Alegre | Primary care coverage areas | 2003 | HIV/Aids | Immunization coverage as a proxy of primary carre unit reach out | |
| Database search | Accessibility of diagnostic and treatment centres for visceral leishmaniasis in Gedaref State, northern Sudan | (Gerstl, Amsalu, and Ritmeijer 2006) | Tropical Medicine & International Health (2006) | no | Qualitative interview | Sudan | Southern Gedaref State | Villages | 2003 | Visceral leishmaniasis | Travel time and travel costs | in hours and days |
| Database search | Designing Equitable Antiretroviral Allocation Strategies in Resource-Constrained Countries | (Wilson and Blower 2005) | PLoS Medicine, 2005 | no | Geospatial resource modelling | South Africa | KwaZulu–Natal | Province | 2004-2005 | HIV/Aids | Allocation of resources to ensure access to medication | |
| Database search | DOTS expansion: will we reach the 2005 targets? | (Veron et al. 2004) | The International Journal of Tuberculosis and Lung Disease (2004) | no | Literature review | Global | - | - | 2005 | Tuberculosis | - | - |
| Database search | Spatial and temporal risk factors for the early detection of Trypanosoma brucei rhodesiense sleeping sickness patients in Tororo and Busia districts, Uganda | (Odiit et al. 2004) | Transactions of the Royal Society of Tropical Medicine and Hygiene (2004) | yes | Retrospective | Uganda | Tororo and Busia districts | Districts | 1987-2001 | Sleeping sickness | Distance to health unit | in km |
| Database search | The Health Sector Gap in the Southern Africa Crisis in 2002/2003 | (Griekspoor et al. 2004). | Disasters. 2004 Dec | no | Literature review | Southern African region | | Multiple countries | 2002-2003 | HIV/Aids | Access to essential drugs | in km |
| Database search | Access to health care facilities of suspected dengue patients in Rio de Janeiro, Brazil | (Freitas et al. 2019) | CIENCIA & SAUDE COLETIVA (2019) | yes | Retrospective ecological study | Brazil | Rio de Janeiro | Neighbourhood level | 2011-2013 | Dengue | Linear distance to health facility | |
| Database search | Disparities in access to diagnosis and care in Blantyre, Malawi, identified through enhanced tuberculosis surveillance and spatial analysis | (MacPherson et al. 2019) | BMC Med (2019) | yes | Cross-sectional study | Malawi | Blantyre | Community health worker boundaries | 2015-2017 | Tuberculosis | Distance to health unit | in meters |
| Database search | Distance sampling for epidemiology: an interactive tool for estimating under-reporting of cases from clinic data | (Nelli et al. 2020) | International journal of health geographics (2020) | yes | Retrospective | Burkina Faso | Komoé district | Villages | 2017 | Malaria | Distance to health facility | in km |
| Database search | Distance to clinic is a barrier to PrEP uptake and visit attendance in a community in rural Uganda | (Mayer et al. 2019) | J Int AIDS Soc (2019) | yes | Cluster-randomized community trial | Uganda | Ruhoko | Household | 2017 | HIV/Aids | Distance and travel time | in km and minutes |
| Database search | Geospatial modeling of microcephaly and zika virus spread patterns in Brazil | (Amaral et al. 2019) | PLoS One (2019) | yes | Retrospective ecological study | Brazil | National | Municipality | 2016 | Zika virus infection | Primary care coverage | |
| Database search | HIV Vertical transmission in Rio de Janeiro, Brazil - does the distance matter? | (Hofer et al. 2019) | AIDS Care (2019) | yes | Retrospective | Brazil | Rio de Janeiro | Household | 1996-2013 | HIV/Aids | Distance to health facility | in km |
| Database search | Profile of hospitalizations in Pediatric Intensive Care Units of the Brazilian Unified Health System in the state of Pernambuco, Brazil | (Mendonça et al. 2019) | CIENCIA & SAUDE COLETIVA | no | Cross-sectional study | Brazil | Pernambuco | Municipality | 2010 | Pediatric intensive care hospitalizations | Distance to health facility | in km |
| Database search | Spatial clustering and socio-demographic determinants of HIV infection in Ethiopia, 2015-2017 | (Gelaw et al. 2019) | Internatial Journal of Infectious Diseases (2019) | yes | Retrospective | Ethiopia | Amhara Region | Districts | 2015-2017 | HIV/Aids | Health facility coverage | % |
| Database search | Spatial clustering of notified tuberculosis in Ethiopia: A nationwide study | (Alene and Clements 2019) | PLoS One (2019) | yes | Retrospective ecological study | Ethiopia | National | Districts | 2016-2017 | Tuberculosis | Not specified | - |
| Database search | Spatial patterns of tuberculosis and HIV co-infection in Ethiopia | (Alene et al. 2019) | PLoS One (2019) | yes | Retrospective ecological study | Ethiopia | National | Districts | 2015-2017 | HIV/Aids/TB | Not specified | - |
| Database search | The Impacts of Residential Location on the Risk of HIV Virologic Failure Among ART Users in Durban, South Africa | (Chen et al. 2019) | AIDS Behav (2019) | yes | Retrospective analysis of case-control study | South Africa | Durban | Residental street or sub-place/main place | 2010-2012 | HIV/Aids | Distance and travel time to health facility | in km and minutes |
| Database search | Treatment-seeking behaviour for febrile illnesses and its implications for malaria control and elimination in Savannakhet Province, Lao PDR (Laos): a mixed method study | (Adhikari et al. 2019) | BMC Health Serv Res (2019) | yes | Cross-sectional mixed methods | Laos | Savannakhet province | Household | 2015 | Febrile illnesses | Distance and travel time to health facility | in km and minutes |
| Database search | Uptake of maternal care and childhood immunization among ethnic minority and Han populations in Sichuan province: a study based on the 2003, 2008 and 2013 health service surveys | (Zhang et al. 2019) | BMC Pregnancy Childbirth (2019) | yes | Retrospective cross sectional | China | Sichuan province | District/county | 2003, 2008, 2013 | Childhood vaccination | Distance to health facility | in km |
| Manual inclusion | Use of Geographically Weighted Poisson Regression to examine the effect of distance on Tuberculosis incidence: A case study in Nam Dinh, Vietnam | (Bui et al. 2018) | PLOS One (2018) | yes | Retrospective | Vietnam | Nam Dinh | Communes | 2012-2015 | Tuberculosis | Distance | in km |
| Manual inclusion | The impact of distance of residence from a peripheral health facility on pediatric health utilisation in rural western Kenya | (Feikin et al. 2009) | Tropical medicine & international health (2009) | yes | Retrospective | Kenya | Asembo | Household | 2003-2004 | Disease burden | Distance | in km |
| Manual inclusion | Healthcare-seeking behavior for infectious diseases in a community in Bangladesh | (Khan et al. 2018) | International Journal of Advanced Medical and Health Research (2018) | yes | Cross-sectional study | Bangladesh | Dumki | Subdistrict | 2017 | Disease burden | Travel time to health facilities | in minutes |
| Manual inclusion | Health-Care Access during the Ebola Virus Epidemic in Liberia | (McQuilkin et al. 2017) | The American journal of tropical medicine and hygiene (2017) | no | Community-based survey | Liberia | National | Communes | 2015 | Ebola | Going to health facility and receiving care | |
| Manual inclusion | The Effect of Distance to Health-Care Facilities on Childhood Mortality in Rural Burkina Faso | (Schoeps et al. 2011) | American journal of epidemiology (2011) | yes | Retrospective | Burkina Faso | Nouna | Health district | 1993-2005 | Child mortality | Travel time | in minutes |
| Manual inclusion | Monitoring the impact of decentralised chronic care services on patient travel time in rural Africa - methods and results in Northern Malawi | (Houben et al. 2012) | International journal of health geographics (2012) | yes | Retrospective | Malawi | Karonga district | Household | 2005-2008 | ART medication | Potential and actual travel time | in hours |
| Database search | Landscapes of healthcare utilization during a dengue fever outbreak in an urban environment of Colombia | (Casas and Delmelle 2019) | Environmental monitoring and assessment (2019) | yes | Retrospective | Colombia | Cali | Point locations | 2010 | Dengue | Travel time | in minutes |
| Database search | Travel time to health facilities in areas of outbreak potential: maps for guiding local preparedness and response | (Hulland et al. 2019) | BMC medicine (2019) | yes | Geospatial modelling | African continent | per country | - | 2018 | Viral hemorrhagic fevers | Travel time | in hours |
| Manual inclusion | The impact of primary health care on malaria morbidity--defining access by disease burden. | (O’Meara et al. 2009) | Tropical Medicine & International Health (2009) | yes | Retrospective | Kenya | Kilifi District | District | 2002-2005 | Malaria | Travel time | in minutes |
| Manual inclusion | Predictors of diarrheal mortality and patterns of caregiver health seeking behavior in in Karachi, Pakistan | (Qamar et al. 2016) | Journal of global health (2016) | yes | Retrospective | Pakistan | Karachi | Field sites | 2009-2010 | Diarrhea | Travel time | in hours |
| Database search | Using healthcare-seeking behaviour to estimate the number of Nipah outbreaks missed by hospital-based surveillance in Bangladesh | (Hegde et al. 2019) | International journal of epidemiology (2019) | yes | Retrospective | Bangladesh | Rajshahi, Rangpur, Faridpur | Districts | 2007 | Nipah | Distance | in km |
| Manual inclusion | The association between travel time to health facilities and childhood vaccine coverage in rural Ethiopia. A community based cross sectional study | (Okwaraji et al. 2012) | BMC public health (2012) | yes | Cross-sectional study | Ethiopia | Dabat district | Kebele | 2010 | Childhood vaccination | Travel time | in minutes |
| Manual inclusion | Inpatient child mortality by travel time to hospital in a rural area of Tanzania. | (Manongi et al. 2014) | Tropical Medicine & International Health (2014) | yes | Retrospective | Tanzania | Teule hospital | Hospital | 2005-2006 | Child mortality | Travel time | in hours |
