# Supplementary material for: The winding road to health: A systematic scoping review on the effect of geographical accessibility to health care on infectious diseases in low- and middle-income countries
Source: PLoS One. 2021 Jan 4;16(1):e0244921. doi: 10.1371/journal.pone.0244921 (PMC7781385; doi:10.1371/journal.pone.0244921)
Supplement: S3 Table — (DOCX) [file pone.0244921.s004.docx]

| **Infectious disease** | **N** | **References** |
| --- | --- | --- |
| HIV/Aids | 14 | (Schaefer et al. 2017; Gerberry et al. 2014; Zulu, Kalipeni, and  Johannes 2014; Yao, Agadjanian, and Murray 2014; Fujita et al. 2012;  Yao et al. 2012; Pharris et al. 2011; Barcellos et al. 2009; Wilson and  Blower 2005; Griekspoor et al. 2004.; Mayer et al. 2019;  Hofer et al. 2019; Gelaw et al. 2019; Chen et al. 2019) |
| Tuberculosis | 8 | (Sullivan, Esmaili, and Cunningham 2017; Shaweno et al. 2017;  Kapwata et al. 2017; Zammarchi, Bartalesi, and Bartoloni 2014;  Veron et al. 2004; MacPherson et al. 2019; Alene and Clements 2019;  Bui et al. 2018) |
| Cholera | 5 | (Bwire et al. 2017; Page et al. 2015; Pezeshki et al. 2012;  Tuite et al. 2011; Penrose et al. 2010) |
| Dengue | 5 | (Casas, Delmelle, and Delmelle 2017;  Telle, Vaguet, Yadav, Lefebvre, Daudé, et al. 2016;  Stewart-Ibarra et al. 2014; Freitas et al. 2019;  Casas and Delmelle 2019) |
| Child mortality | 4 | (Sartorius et al. 2010; Ombok et al. 2010; Schoeps et al. 2011;  Manongi et al. 2014) |
| Disease burden | 4 | (Mboera et al. 2014; Etyang et al. 2014; Feikin et al. 2009;  Khan et al. 2018) |
| Childhood vaccination | 3 | (Blanford et al. 2012; Zhang et al. 2019; Okwaraji et al. 2012) |
| Malaria | 3 | (Minale and Alemu 2018; Nelli et al. 2020; O’Meara et al. 2009) |
| HIV/Aids/TB | 2 | (Mee et al. 2014; Alene et al. 2019) |
| Measles | 2 | (Rivadeneira, Bassanesi, and Fuchs 2018; Poletti et al. 2018) |
| ART medication | 1 | (Houben et al. 2012) |
| Bacillary dysentery | 1 | (Nie et al. 2014) |
| Diarrhea | 1 | (Qamar et al. 2016) |
| Dog-bites | 1 | (Zaidi et al. 2013) |
| Ebola | 1 | (McQuilkin et al. 2017) |
| Febrile illnesses | 1 | (Adhikari et al. 2019) |
| Nipah | 1 | (Hegde et al. 2019) |
| Pediatric intensive care  hospitalizations | 1 | (Mendonça et al. 2019) |
| Sleeping sickness | 1 | (Odiit et al. 2004) |
| Tetanus | 1 | (Sutiono et al. 2009) |
| Typhoid fever | 1 | (Brijnath and De Souza 2012) |
| Viral hemorrhagic fevers | 1 | (Hulland et al. 2019) |
| Visceral leishmaniasis | 1 | (Gerstl, Amsalu, and Ritmeijer 2006) |
| Zika virus infection | 1 | (Amaral et al. 2019) |

### **S3 Table. Number of papers per disease category**
