# Supplementary material for: The winding road to health: A systematic scoping review on the effect of geographical accessibility to health care on infectious diseases in low- and middle-income countries
Source: PLoS One. 2021 Jan 4;16(1):e0244921. doi: 10.1371/journal.pone.0244921 (PMC7781385; doi:10.1371/journal.pone.0244921)
Supplement: S4 Table — (DOCX) [file pone.0244921.s005.docx]

| **Country** | **n** | **Author** |
| --- | --- | --- |
| Ethiopia | 7 | (Minale and Alemu 2018; Shaweno et al. 2017; Poletti et al. 2018;  Gelaw et al. 2019; Alene and Clements 2019; Alene et al. 2019;  Okwaraji et al. 2012) |
| South Africa | 6 | (Kapwata et al. 2017; Gerberry et al. 2014; Mee et al. 2014; Sartorius et al. 2010;  Wilson and Blower 2005; Chen et al. 2019) |
| Brazil | 5 | (Barcellos et al. 2009; Freitas et al. 2019; Amaral et al. 2019; Hofer et al. 2019;  Mendonça et al. 2019) |
| Kenya | 4 | (Etyang et al. 2014; Ombok et al. 2010; Feikin et al. 2009; O’Meara et al. 2009) |
| Global | 3 | (Zammarchi, Bartalesi, and Bartoloni 2014; Brijnath and De Souza 2012;  Veron et al. 2004) |
| Malawi | 3 | (Zulu, Kalipeni, and Johannes 2014; MacPherson et al. 2019;  Houben et al. 2012) |
| Uganda | 3 | (Bwire et al. 2017; Odiit et al. 2004; Mayer et al. 2019) |
| Vietnam | 3 | (Fujita et al. 2012; Pharris et al. 2011; Bui et al. 2018) |
| Bangladesh | 2 | (Khan et al. 2018; Hegde et al. 2019) |
| Burkina Faso | 2 | (Nelli et al. 2020; Schoeps et al. 2011) |
| China | 2 | (Nie et al. 2014; Zhang et al. 2019) |
| Colombia | 2 | (Casas, Delmelle, and Delmelle 2017; Casas and Delmelle 2019) |
| Ecuador | 2 | (Rivadeneira, Bassanesi, and Fuchs 2018; Stewart-Ibarra et al. 2014) |
| Haiti | 2 | (Page et al. 2015; Tuite et al. 2011) |
| Mozambique | 2 | (Yao, Agadjanian, and Murray 2014; Yao et al. 2012) |
| Pakistan | 2 | (Zaidi et al. 2013; Qamar et al. 2016) |
| Sub-Saharan  Africa | 2 | (Sullivan, Esmaili, and Cunningham 2017; Mboera et al. 2014) |
| Tanzania | 2 | (Penrose et al. 2010; Manongi et al. 2014) |
| African  continent | 1 | (Hulland et al. 2019) |
| India | 1 | (Telle, Vaguet, Yadav, Lefebvre, Daudé, et al. 2016) |
| Indonesia | 1 | (Sutiono et al. 2009) |
| Iran | 1 | (Pezeshki et al. 2012) |
| Laos | 1 | (Adhikari et al. 2019) |
| Liberia | 1 | (McQuilkin et al. 2017) |
| Niger | 1 | (Blanford et al. 2012) |
| Southern  African region | 1 | (Griekspoor et al. 2004.) |
| Sudan | 1 | (Gerstl, Amsalu, and Ritmeijer 2006) |
| Zimbabwe | 1 | (Schaefer et al. 2017) |

### **S4 Table. Number of papers per country where studies were conducted**
