# Supplementary material for: The winding road to health: A systematic scoping review on the effect of geographical accessibility to health care on infectious diseases in low- and middle-income countries
Source: PLoS One. 2021 Jan 4;16(1):e0244921. doi: 10.1371/journal.pone.0244921 (PMC7781385; doi:10.1371/journal.pone.0244921)
Supplement: S1 File — (DOCX) [file pone.0244921.s006.docx]

**S1 File. Database search terms.**

**PubMed search term:**

((((access*[Title/Abstract] OR distance*[Title/Abstract] OR "travel time"[Title/Abstract] OR "barriers to access"[Title/Abstract] OR "access barriers")[Title/Abstract] AND (geographic*[Title/Abstract] OR geospatial[Title/Abstract] OR GIS[Title/Abstract] OR "geo* information system"[Title/Abstract] OR spatial)[Title/Abstract] AND ((health AND(facilit*[Title/Abstract] OR cent*[Title/Abstract] OR care))[Title/Abstract] OR hospital)[Title/Abstract] AND (epidemic[Title/Abstract] OR outbreak[Title/Abstract] OR infectious disease*))[Title/Abstract])) OR ((("Health Services Accessibility"[Mesh]) AND ("Disease Outbreaks"[Mesh] OR "Epidemics"[Mesh]) AND ("Geographic Information Systems"[Mesh])))

**Web of Science search term:**

TOPIC: (((access* OR distance* OR "travel time" OR "barriers to access" OR "access barriers") AND (geographic* OR geospatial OR GIS OR "geo* information system" OR spatial) AND ((health AND (facilit* or cent* or care)) OR hospital) AND (epidemic OR outbreak OR infectious disease*)))
